# Exposure to US Cancer Drugs With Lack of Confirmed Benefit After US Food and Drug Administration Accelerated Approval

**DOI:** 10.1001/jamaoncol.2022.7770

**Published:** 2023-02-23

**Authors:** Ravi B. Parikh, Rebecca A. Hubbard, Erkuan Wang, Trevor J. Royce, Aaron B. Cohen, Amy S. Clark, Ronac Mamtani

**Affiliations:** 1 Perelman School of Medicine, University of Pennsylvania, Philadelphia, Pennsylvania; 2 Flatiron Health, New York, New York

## Abstract

This cross-sectional study evaluates patient exposure to oncology drugs withdrawn from the US Food and Drug Administration (FDA) Accelerated Approval program.

Between 2009 and 2022, the US Food and Drug Administration (FDA) approved 48 drugs for 66 oncology-related indications under the Accelerated Approval (AA) program.^1^ Indications granted AA based on surrogate end points are subsequently required to confirm clinical benefit.^2^ Since 2009, 15 indications (23%) have been withdrawn due to lack of benefit over standard of care.^3^ We estimate the proportion of patients who received treatment for 5 oncology-related indications later withdrawn after failure to confirm efficacy under the AA program.

## Methods

This cross-sectional study included patients with advanced or recurrent breast, bladder, hepatocellular, gastric, or small cell lung cancer between May 18, 2016, and March 8, 2022, treated with at least 1 line of systemic therapy. We used deidentified, electronic health record (EHR)–derived, patient-level data curated via technology-enabled abstraction.^4^ The Copernicus Group and University of Pennsylvania institutional review boards approved this study and granted waivers of informed consent owing to use of deidentified data. This study followed the STROBE reporting guideline.

We studied 5 disease-specific AA indications with subsequent published negative phase III confirmatory trials and indication withdrawal (Table). These drugs had additional biomarker-specific, cancer-agnostic indication approvals, such that they were available for use before AA and continued to be available after AA withdrawal. Outcomes were calculated for patients aged 18 years or older who initiated therapy and were eligible for the AA indication. Race and ethnicity were identified from the EHR and included as covariates because minority status is associated with decreased access to novel therapies. We excluded patients with 1 or more cancers or, for biomarker-specific indications, missing biomarker data. The primary outcome was initiation of AA therapies as a proportion of all indication-specific treatment initiations. We calculated the primary outcome at 3 intervals: AA to indication withdrawal, AA to negative confirmatory trial, and negative trial to indication withdrawal. Because 4 indications were reviewed at the April 2021 FDA Oncology Drug Advisory Committee meeting, we included this date as a discrete point. Logistic regression–estimated time trends with restricted cubic splines were used to estimate the daily percentage of treatment-eligible patients who initiated a withdrawn AA drug. Analyses were performed using SAS, version 9.4 (SAS Institute Inc).

**Table.** Indications, Dates, and Observed Prevalence of Accelerated Approval (AA) Drug Usage

	Atezolizumab	Pembrolizumab	Atezolizumab	Nivolumab	Nivolumab
Indication^a^	Breast, triple-negative	Gastric, PD-L1 positive	Bladder	HCC	SCLC
Line of therapy	First	Third	Later	Later	Later
Date of AA	3/8/2019	9/22/2017	5/18/2016	9/22/2017	8/16/2018
Date of negative trial publication	7/1/2021	6/4/2018	2/24/2018	10/1/2019	5/1/2021
Date of withdrawal	9/25/2021	7/7/2021	3/8/2021	7/23/2021	12/29/2020
Prevalence per interval, %					
AA to withdrawal	23.1	41.4	22.5	38.8	23.6
AA to negative confirmatory trial	24.1	71.4	39.7	55.6	21.0
Negative confirmatory trial to withdrawal	8.6	38.6	12.8	20.3	2.5

Abbreviations: HCC, hepatocellular carcinoma; PD-L1, programmed cell death 1 ligand 1; SCLC, small cell lung cancer.

^a^ All indications are for advanced cancer.

## Results

The cohort included 4342 patients who received 6560 eligible lines of therapy (median age, 70 [range, 24-85] years; female, 1639 [39%]; male, 2649 [61%]; Asian, 93 [2%]; Black, 348 [8%]; Hispanic, 248 [6%]; White, 2909 [67%]; Other, 637 [15%]), and 3709 (85%) received care at community practices. The median time from AA to indication withdrawal was 46 (range, 12-58) months. Between AA and subsequent withdrawal, 1361 oncology treatment initiations (26.1%) involved an AA therapy that was subsequently withdrawn (triple-negative breast, 23.1% [113 of 490]; bladder, 22.5% [695 of 3096]; hepatocellular, 38.8% [323 of 832]; gastric, 41.4% [101 of 244]; small cell lung, 23.6% [129 of 546]) (breast and bladder shown in the Figure). Prevalence of AA drug initiations was higher between AA and negative trial publication (overall, 35.5%; triple-negative breast, 24.1%; bladder, 39.7%; hepatocellular, 55.6%; gastric, 71.4%; small cell lung, 21.0%) than between negative trial publication and withdrawal (15.7%, 8.6%, 12.8%, 20.3%, 38.6%, 2.5%, respectively) (Table).

**Figure.**
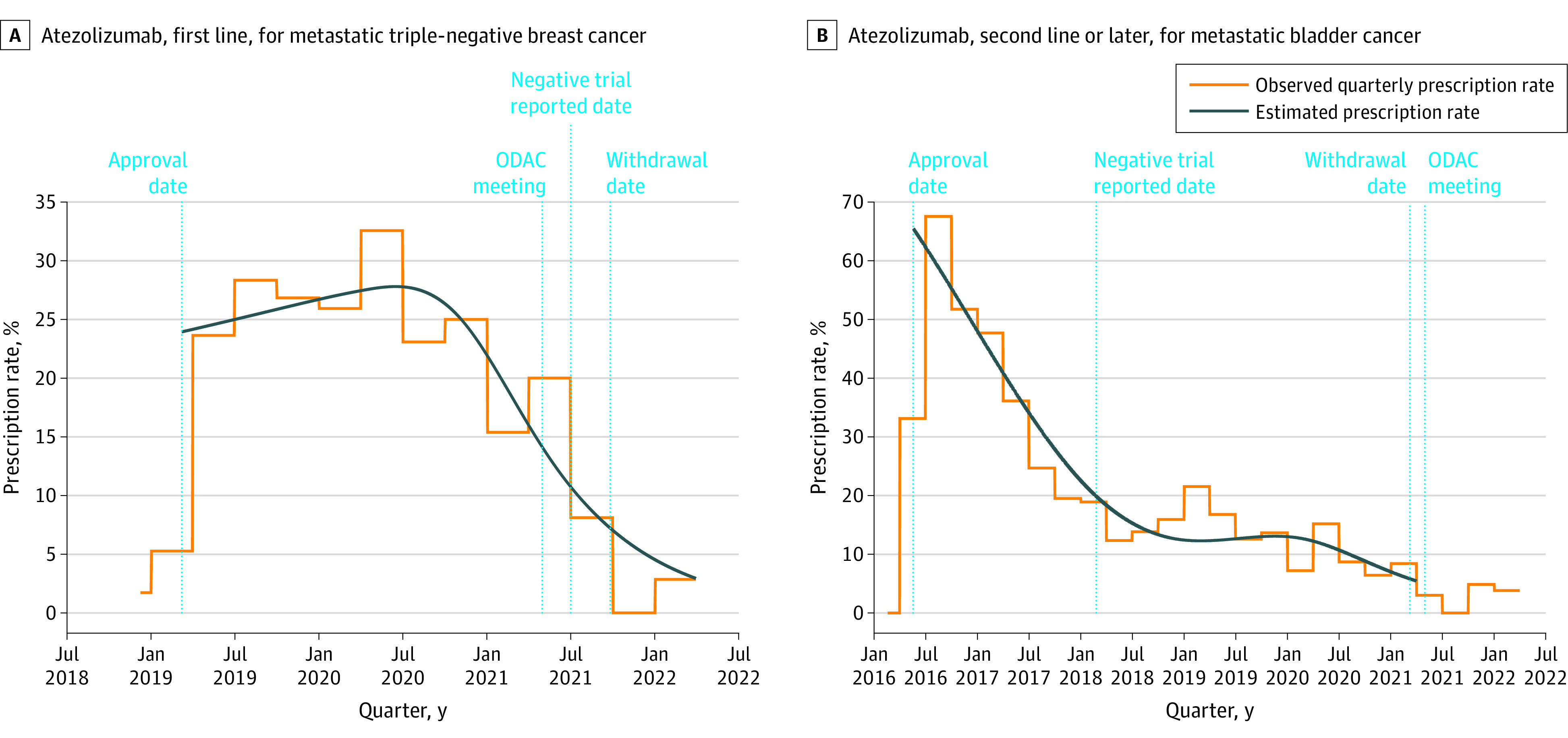
Representative Trends in Accelerated Approval (AA) Oncology Drug Use Over Time, 2016-2022 Shown are the trends in use of oncology drugs that received AA and were subsequently withdrawn between 2016 and 2022. Observed quarterly prescription rate of AA therapies is a proportion of all treatment initiations for an indication, and estimated prescription rate is the daily percentage of treatment-eligible patients who used an AA drug based on logistic regression with restricted cubic splines. ODAC indicates the US Food and Drug Administration Oncology Drug Advisory Committee.

## Discussion

Among 5 oncology indications, 26.1% of eligible treatment initiations involved an AA indication subsequently withdrawn due to lack of benefit. An expected trade-off exists between expediting access to promising cancer drugs and withdrawal of some indications.^5^ Given the growth of withdrawals due to negative confirmatory trials and emerging evidence on the high spending associated with AA drugs, it is critical to balance early access against population-level exposure to cancer therapies with no benefit over standard of care.^6^ Limitations included an inability to assess population-level exposure because only 5 withdrawn AA indications had sufficient sample and follow-up for analysis. Earlier access and more rapid FDA responses to negative confirmatory trial data, a key proposal of the Accelerated Approval Integrity Act proposed in March 2022, may minimize exposure to AA therapies with lack of benefit.

## Notes

Supplement.
Data Sharing Statement
