# Supplementary material for: Exposure to US Cancer Drugs With Lack of Confirmed Benefit After US Food and Drug Administration Accelerated Approval
Source: JAMA Oncol. 2023 Feb 23;9(4):567–9. doi: 10.1001/jamaoncol.2022.7770 (PMC9951100; doi:10.1001/jamaoncol.2022.7770)
Supplement: Supplement. — Data Sharing Statement [file jamaoncol-e227770-s001.pdf]

Data Sharing Statement

Parikh. Exposure to US Cancer Drugs With Lack of Confirmed Benefit After US Food and Drug Administration Accelerated Approval. *JAMA Oncol*. Published February 23, 2023. doi:10.1001/jamaoncol.2022.7770

Data
Data available: Yes
Data types: Deidentified participant data, Data dictionary
How to access data: Email ravi.parikh@pennmedicine.upenn.edu with requests for data
When available: With publication

Supporting Documents
Document types: None

Additional Information
Who can access the data: Researchers whose proposed use of the data has been approved
Types of analyses: For a research purpose
Mechanisms of data availability: after approval of a proposal and with a signed data access agreement
